# Outbreak of *Ralstonia mannitolilytica* bacteraemia in patients undergoing haemodialysis at a tertiary hospital in Pretoria, South Africa

**DOI:** 10.1186/s13756-020-00778-7

**Published:** 2020-07-29

**Authors:** Mohamed Said, Wesley van Hougenhouck-Tulleken, Rashmika Naidoo, Nontombi Mbelle, Farzana Ismail

**Affiliations:** 1 Department of Medical Microbiology, University of Pretoria, Pathology Buiding, Prinshof Campus, Room 3-22, 5 Bopelo Road, Pretoria, South Africa; 2 Division of Nephrology, Steve Biko Academic Hospital, Steve Biko Road &, Malan St, Prinshof 349-Jr, Pretoria, South Africa; 3 Department of Internal Medicine, University of Pretoria, Steve Biko Road &, Malan St, Prinshof 349-Jr, Pretoria, South Africa; 4 National Health Laboratory Services, Tshwane Academic Division, 5 Bopelo Road, Riviera, Pretoria, South Africa; 5 Centre for Tuberculosis, National Institute for Communicable Disease, 1 Modderfontein Road, Sandringham, Johannesburg, South Africa

**Keywords:** *Ralstonia mannitolilytica*, Outbreak, Hospital environment, Healthcare associated infections, Haemodialysis unit, Dialysis water, Culture, Molecular confirmation

## Abstract

**Background:**

*Ralstonia* species are Gram-negative bacilli of low virulence. These organisms are capable of causing healthcare associated infections through contaminated solutions. In this study, we aimed to determine the source of *Ralstonia mannitolilytica* bacteraemia in affected patients in a haemodialysis unit.

**Methods:**

Our laboratory noted an increase in cases of bacteraemia caused by *Ralstonia mannitililytica* between May and June 2016. All affected patients underwent haemodialysis at the haemodialysis unit of an academic hospital. The reverse osmosis filter of the haemodialysis water system was found to be dysfunctional. We collected water for culture at various points of the dialysis system to determine the source of the organism implicated. ERIC-PCR was used to determine relatedness of patient and environmental isolates.

**Results:**

Sixteen patients were found to have *Ralstonia mannitolilytica* bacteraemia during the outbreak period. We cultured *Ralstonia* spp. from water collected in the dialysis system. This isolate and patient isolates were found to have the identical molecular banding pattern.

**Conclusions:**

All patients were septic and received directed antibiotic therapy. There was 1 mortality. The source of the *R. mannitolilytica* infection in these patients was most likely the dialysis water as the identical organism was cultured from the dialysis water and the patients. The hospital management intervened and repaired the dialysis water system following which no further cases *of R. mannitolilytca* infections were detected. A multidisciplinary approach is required to control healthcare associated infections such as these. Routine maintenance of water systems in the hospital is essential to prevent clinical infections with *R.mannitolilytica*.

## Background

*Ralstonia* species are Gram-negative non-fermentative rods which are environmental organisms commonly found in soil, water and plants [1]. These bacteria are generally of low virulence and are therefore regarded as opportunistic pathogens causing disease in immunocompromised individuals [2]. Infections caused by *Ralstonia* species include sepsis, meningitis and central venous catheter associated bacteraemia [1]. These organisms are also capable of causing hospital outbreaks associated with contaminated solutions including water for injection, saline solutions, disinfectants and antiseptics [2]. In addition, *Ralstonia* species have a tendency to form biofilm which enhances the organisms survival in the environment (including the hospital environment), and likely plays a role in their frequent antibiotic resistance [3].

The most commonly reported *Ralstonia* species clinically is *Ralstonia pickettii* [2]. Clinical infections however can also be caused by *Ralstonia mannitolilytica* and *Ralstonia insidiosa* [2].

Currently, there are no clear treatment guidelines for *R. mannitolilytica* infections. In addition, there are no Clinical and Laboratory Standards Institute (CLSI) breakpoints developed for *Ralstonia* species. Treatment is challenging as this species is frequently resistant to many antibiotics [2]. *Ralstonia* species are generally resistant to many of the β-lactam class of antibiotics, including the carbapenems. Class D carabapenmase genes such as *bla*_OXA-22_ and *bla*_OXA-60_ are commonly associated with *Ralstonia* species [4]. Treatment of *Ralstonia* infection is usually based on the susceptibility profile of the organism isolated [2].

In this study, we aimed to determine the source of *Ralstonia mannitolilytica* bacteraemia in affected patients in a haemodialysis unit.

## Methods

### Aim

In this study, we aimed to determine the source of *Ralstonia mannitolilytica* bacteraemia in affected patients in a haemodialysis unit.

### Study design and setting

The Tshwane Academic Division Microbiology laboratory in Pretoria, South Africa detected an unusual increase in the number of bacteraemia cases with *Ralstonia mannitolillytica* between May and June 2016. Sixteen patients in total were found to have bloodstream infections with this organism. The isolates were identified using the Vitek 2 (Biomerieux, France) identification system. The identity of the organism was confirmed by 16S rRNA sequencing which was done for the first 4 *R. mannitolillytica* isolates cultured. Antimicrobial susceptibility testing (AST) was performed using the Kirby-Bauer disc diffusion method.

A line list was drawn up of the 16 cases detected. Although the laboratory serves a number of hospitals in the Pretoria region, all patients with *R. mannitolillytica* infections were from the Steve Biko Academic Hospital, suggesting a localised problem to one hospital. All patients were noted to also having undergone haemodialysis at the hospital, which is a water intensive procedure.

**Fig. 1:**
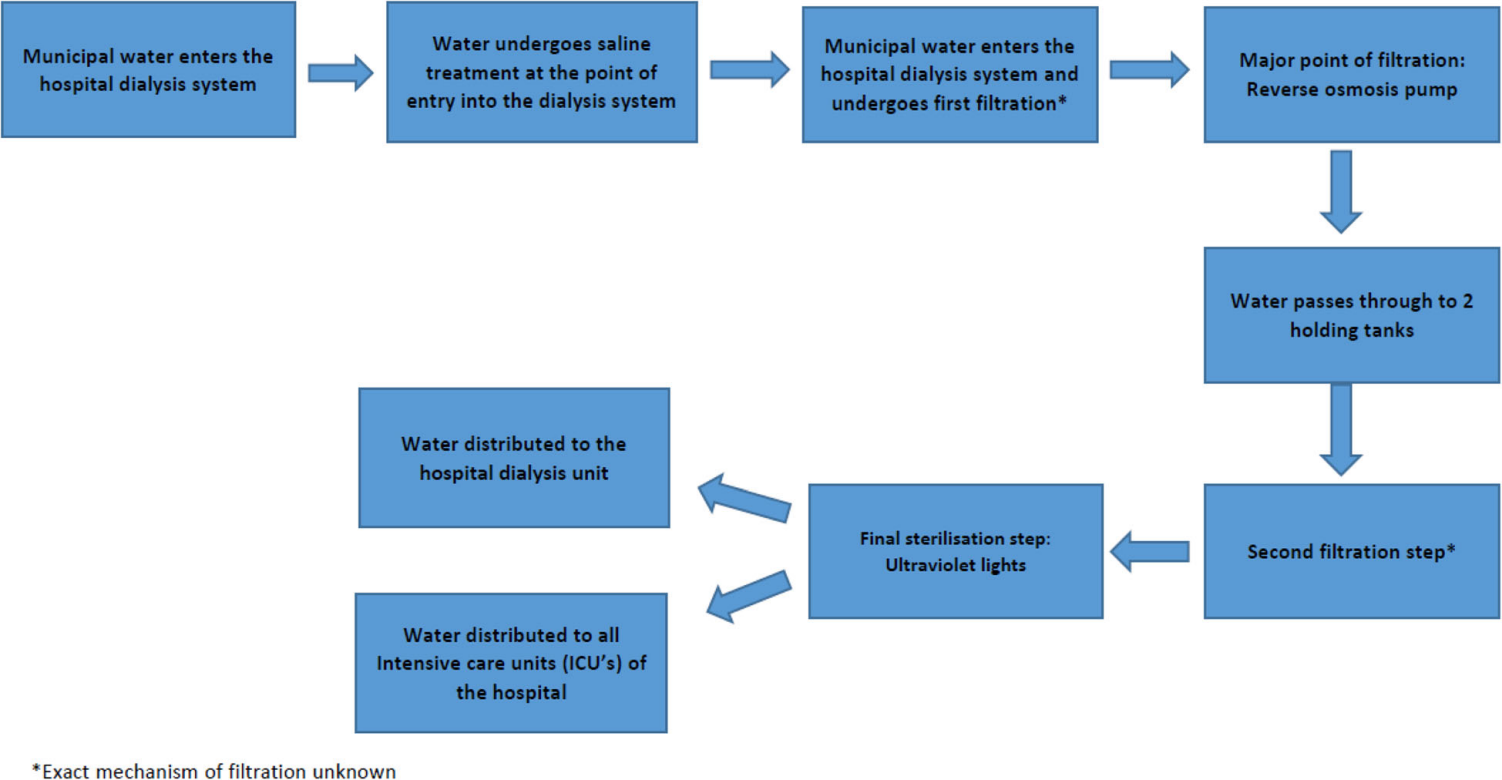
Flow diagram depicting water purification steps prior to the water reaching the hospital

The haemodialysis unit of the Steve Biko Hospital was visited and upon enquiry it was reported that the dialysis water system was faulty. Water entering the hospital is diverted to the dialysis purification conduit, where it passes through a number of filters. Thereafter the water passes through a reverse osmosis pump which is the main point of purification of the dialysis water. Following this, the water then passes through 2 UV (ultraviolet) lights and then into the haemodialysis unit. The reverse osmosis pump was visibly dysfunctional with leaks noted around the pump (Fig. 1). It was hypothesized at this point that contaminated water in the dialysis system was the most likely source of the patients’ bacteraemia. In order to confirm this, we sought to collect water samples from various points of the dialysis purification system as follows.

### Sample collection and processing

**Fig. 2:**
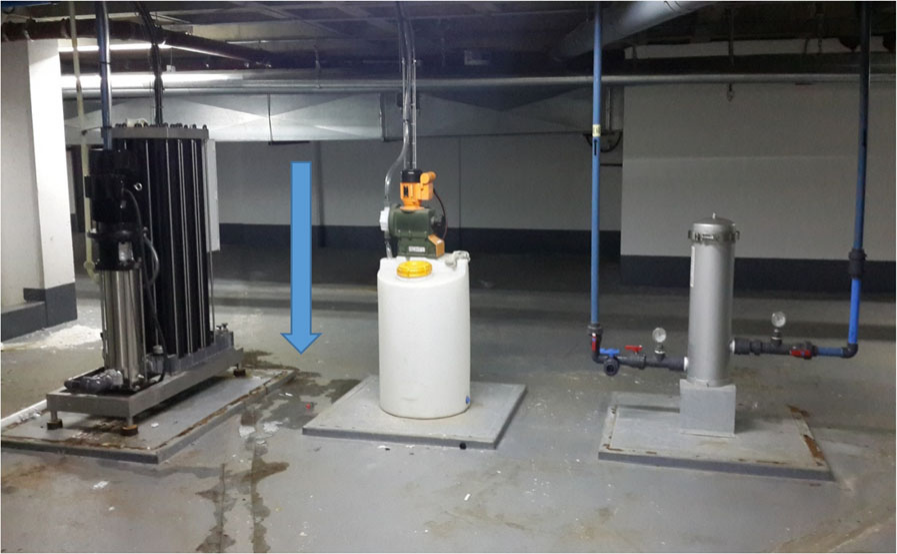
Series of filters and reverse osmosis pump (right to left). Note the leak noted around the reverse osmosis pump (blue arrow)

**Fig. 3:**
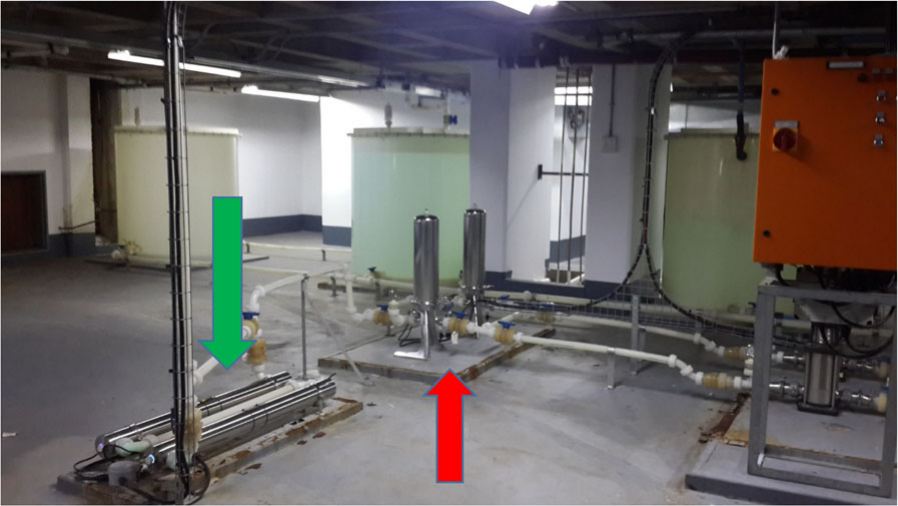
Series of filters (red arrow) past the reverse osmosis pump and 2 UV lights (green arrow) which is the final sterilisation step prior to water entering the wards

Water was collected for testing from a number of points from the dialysis purifications system. Figure 1 shows a flow diagram of the water purification steps of the dialysis water at the Steve Biko Academic Hospital. We collected water at the following points in the system:

- The point of entry into the purification system but before passing through the reverse osmosis pump (Fig. 2).
- After the reverse osmosis pump and before passing through the UV light (Fig. 3).
- From the haemodialysis unit after the water had passed through the UV light source.

Ten millilitres of all the water samples collected were inoculated into aerobic blood culture bottles (BacT/ALERT®) and incubated in the BacT/ALERT® 3D instruments (bioMerieux, France). Once flagged positive, a Gram stain was done on the bottle and the broth was plated onto 5% sheep blood agar, chocolate agar and MaCconkey agar. The plates were incubated at 35-37 °C in ambient air. Plates were inspected for growth at 24 h and again at 48 h. The cultured colonies were identified using the Vitek 2 automated identification system (bioMerieux, France). Antimicrobial susceptibility testing was performed using the Kirby-Bauer disc diffusion method.

Due to limited resources, Enterobacterial Repetitive Intergenic Consensus Polymerase Chain Reaction (ERIC-PCR) was performed on the 16 isolates which were stored during this outbreak to determine their genetic relatedness. The primers used for ERIC-PCR were synthesized by Inqaba Biotechnical Industries (Pty) Ltd., Pretoria, South Africa. The primers were prepared according to the manufacturer’s instructions and PCR set up was according to the Bioline kit (Bioline Reagents Limited, London, UK). The oligonucleotide primer sequences for determining the genetic relatedness are shown in Table 1. It should be noted that the primers described are actually for the molecular typing of *Pseudomonas aeruginosa*. *Ralstonia* species were reclassified from *Pseudomonas* rRNA group II [6]. These organisms are phylogenically very similar [6]. Therefore, the use of the same primers was justified.

**Table 1:** Oligonucleotide primer sequences of the primers used for ERIC-PCR assays for the molecular typing of *Pseudomonas aeruginosa* isolates

Primers	Primer Sequence (5′------------3′)	Product size	Reference
ERIC-1R	CACTTAGGGGTCCTCGAATGTA	N/A	[5]
ERIC-2	AAGTAAGTGACTGGGGTGAGCG		

*N/A* not applicable, *F* sense primer, *R* antisense primer

The preparation of the reaction composition was modified according to the recommendation of the master mix manufacturer (Bioline, London, United Kingdom). The PCR master mix contained the HotStar*Taq* DNA polymerase supplied in the Bioline PCR buffer containing 6 mM MgCl2 (pH 8.7) and a dNTP mix (Bioline, London, United Kingdom).

Each 25 μl reaction mixture contained HotStar*Taq* DNA polymerase, 10× primer mix, RNase free water and template DNA, using 2 μL template DNA (< 1 μg DNA/25 μL). The PCR amplification conditions were; initial denaturation at 95 °C for 15 s, denaturation at 95 °C for 10 s for 45 cycles, annealing at 65 °C for 10 s, extension at 72 °C for 20 s and final extension at 72 °C for 5 min [7]. A negative control (no template) and positive control (*Pseudomonas aeruginosa* ATCC 27853) were included in all PCR assays. The PCR reaction mixture was amplified using the G-Storm thermocycler (G-storm Ltd., Somerton, UK) by means of PCR thermocycling. A 100 bp Plus ladder (ThermoScientific, USA) was used as a reference standard on the 1.8% (m/v) agarose gel that was run at 100 V for 3 h. The ERIC PCR assay gel was viewed using the transilluminator (DigiDoc-It, UVP, LCC, USA). The image of the ERIC-PCR assay gel was stored and analysed using GelCompar II (Applied Maths, Belgium) software.

## Results

In total 16 patients were found to have *Ralstonia mannitolilytica* bacteraemia during the period of the outbreak, which lasted about 2 months (Table 2). The median age of infected patients was 39 years. The majority of the infected patients were male (69%). The mortality rate during the outbreak was 6%.

**Table 2:** Patient information

Patient	Age	Sex	Demised during *Ralstonia* Outbreak	First negative follow up culture	Days to negative culture
1	47	F	No	2016/06/13	33
2	18	M	No	2016/06/26	34
3	22	F	Yes	2016/05/31	2
4	47	M	No	2016/06/05	5
5	37	M	No	2016/06/08	4
6	48	M	No	2016/06/14	5
7	55	M	No	2016/06/16	2
8	47	M	No	2016/06/25	11
9	33	M	No	2016/07/20	34
10	27	M	No	2016/07/17	18
11	28	F	No	2016/07/13	5
12	47	F	No	2016/07/15	2
13	53	M	No	2016/07/19	5
14	45	F	No	2016/08/13	34
15	41	M	No	2016/07/27	7
16	29	M	No	2016/08/02	4

The mean time to the first negative subsequent culture was 13 days.

All isolates in this study had identical antimicrobial susceptibility patterns and were resistant to amoxicillin clavulanic acid, ceftazidime, ertapenem, meropenem, tobramycin, aztreonam and colistin. There were no zones of inhibition and growth of the test organism was noted up to the antibiotic disc. The isolates appeared susceptible to the following antibiotics, with the zone sizes documented in brackets: piperacillin tazobactam (30 mm), ciprofloxacin (30 mm), levofloxacin (28 mm), co-trimoxazole (> 30 mm),, ceftriaxone (> 30 mm), cefuroxime (> 30 mm), cefepime (> 30 mm) and imipenem (33 mm). There are no CLSI clinical breakpoints for *Ralstonia* species. We therefore interpreted the zones based on the breakpoints for *Pseudomonas aeruginosa* from the CLSI M-1002016 document.

The organisms cultured from various points of collection along the dialysis purification system are summarised in Table 3.

**Table 3:** Organisms cultured from dialysis water

Point of collection	Organism/s cultured
At the point of entry into system, before the reverse osmosis pump	1. *Bacillus* spp. 2. *Stenotrophomonas maltophila*
After the reverse osmosis pump, before being subject to UV	1. *Ralstonia pickettii* 2. *Sphingomonas paucimobilis* 3. *Cupriavadus pauculus*
After passing through UV light, from the haemodialysis unit	1. *Sphingomonas paucimobilis*

**Fig. 4:**
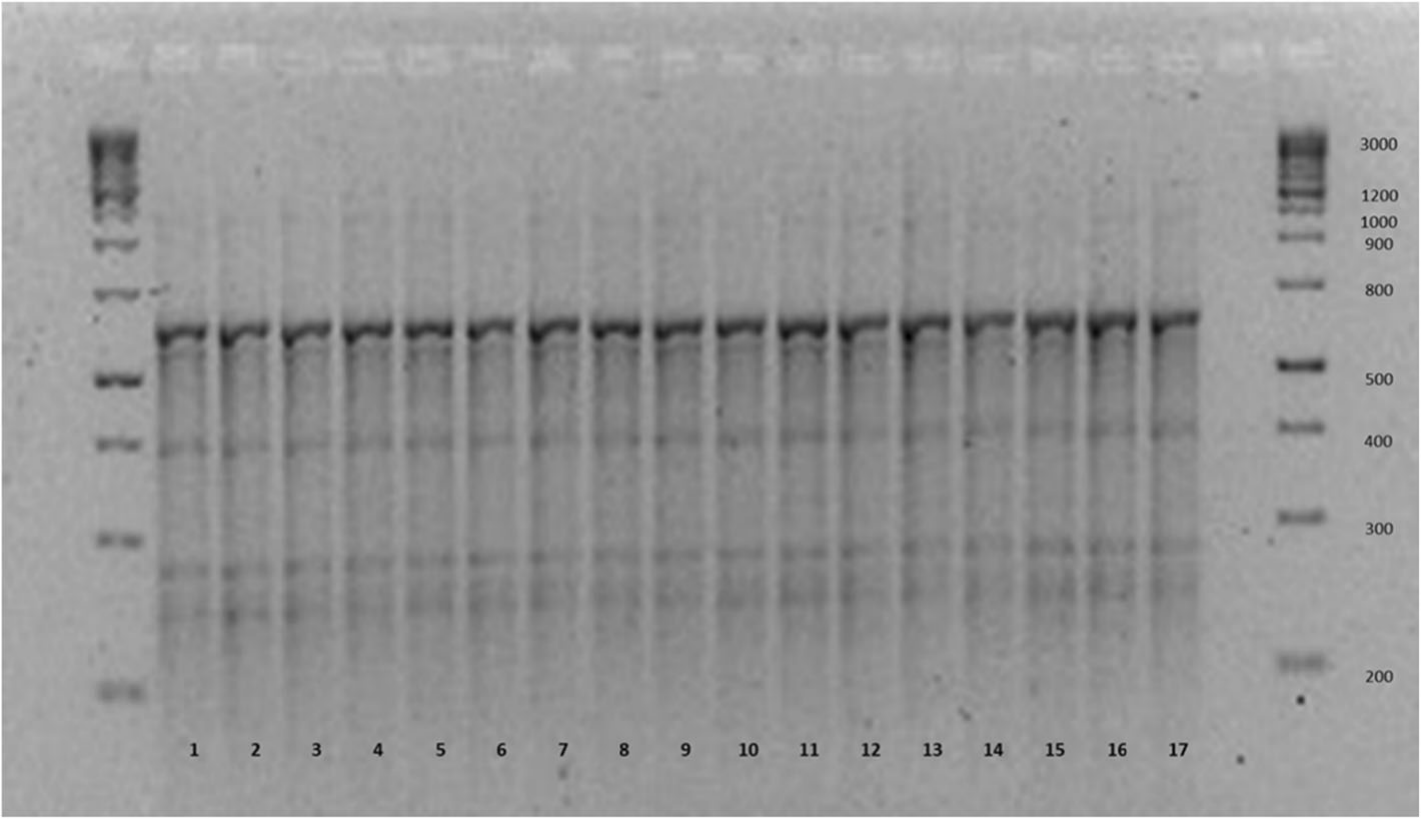
ERIC PCR gel results of *Ralstonia* isolates cultured

ERIC-PCR revealed that all clinical *Ralstonia mannitolilytica* isolates were identical (Fig. 1, lanes 1–16). The *Ralstonia pickettii* isolate cultured from water which had passed the reverse osmosis pump also showed the identical banding on ERIC-PCR (Fig. 4, lane 17). The *Ralstonia* species cultured from water was most likely misidentified by the Vitek 2 system as *R.pickettii*. Species level identification is not always accurate with automated identification systems such as Vitek 2.

## Discussion

Sixteen cases of *Ralstonia mannitolilytica* bacteraemia were detected during the outbreak in the haemodialysis unit at the Steve Biko hospital. Prior to the outbreak, we had no reported isolates of this organism being cultured over the previous year.

All patients presented with fever and rigours whilst undergoing haemodialysis, usually within the first hour of dialysis. The usual patient profile was a patient with an indwelling haemodialysis catheter (no arterio-venous fistulae patients became septic) who had end stage renal disease, complicated by hypertension and mineral bone disease. Empiric antibiotic cover was started with vancomycin and a carbapenem (imipenem or meropenem), as per Steve Biko Academic Nephrology guidelines for line sepsis. This was de-escalated according to the culture and antimicrobial susceptibility results as they became available. In this case, de-escalation to piperacillin-tazobactam was advised if the clinicians decided to continue therapy as all isolates were susceptible to this antibiotic.

All sixteen patients survived except one, who demised after a long hospital stay and many complications. Whilst the mortality was unlikely due to *Ralstonia* sepsis, the precipitating event was *Ralstonia* septicaemia. All patients had their dialysis catheters changed, barring one patient who declined the surgery. This patient was given a 2 week course of high dose imipenem whilst on dialysis (i.e. every second day), and the dialysis catheter was locked with imipenem after dialysis. This resulted in good recovery for the patient.

The mean time to the first negative subsequent culture was 13 days in these patients. It should be noted however that during the course of the outbreak, the same water system was used to administer dialysis to these patients. This could have impacted on the duration of blood culture positivity and response to therapy in these patients.

*R.mannitolilytica* is frequently recognised as a multidrug resistant organism [8]. Daxboeck et al. reported carbapenem resistance in 12 out of their 30 strains [9]. In an Italian study of *R.mannitolilytica* isolates, no carbapenemase genes were detected but all isolates had phenotypic evidence of AmpC β-lactamases [1]. These enzymes confer a wide resistance to many β-lactam agents such as the cephalosporins, β-lactam- β-lactamase-inhibitor combinations, aztreonam and carbapenems, in case of association with altered porins and/or efflux mechanisms [10]. All isolates in this study were resistant to meropenem, but susceptible to imipenem. Sequencing for detection of resistance genes was not performed.

Environmental organisms, namely *Bacillus* species and *Stenotrophomonas* were recovered from water prior to it entering the reverse osmosis pump however these organisms were not found after this point. The reverse osmosis pump probably had some residual effect against these organisms. However, a number of hydrophilic organisms were cultured from water collected after it had passed through the reverse osmosis pump. The contamination at this point may have been through leaks in the reverse osmosis pump which were noted and visible in Fig. 2. The water then passed through the UV light which significantly reduced the numbers of *Cupriavadus pauculus* and *Ralstonia pickettii* organisms such that they were not cultured after passage through UV light. *Sphingomonas paucimobilis* seemed unaffected by UV light and was still cultured after this point although the organism was never cultured from clinical samples. We postulated that *Ralstonia* species may have survived the UV light and occurred in small numbers, likely below the limit of detection of culture, hence the reason that it was cultured from patient specimens but not from the water samples collected in the wards. An alternative explanation could be that UV treatment resulted in the unculturable state of *Ralstonia* species, and the host environment with poor immunity would resuscitate these unculturable bacteria.

Laboratory identification may be problematic with *Ralstonia* species*. Ralstonia mannitolilytica* shows similar biochemical properties to *Ralstonia pickettii* [3]. Tests for nitrate reduction (negative in *R.mannitolilytica*) and acidification of D-arabitol and mannitol (both negative in *R.pickettii*) can differentiate the 2 species [11]. Matrix assisted laser desorption and ionisation time of flight methods such as MALDI TOF MS (Biomerieux, France) has also been found to yield a more accurate species level identification of *Ralstonia* species [4]. 16S rRNA gene sequencing has proved very useful in the definitive identification of *R.mannitolilytica*, and is now considered the reference method for identification to species level [4, 12]. We used the Vitek 2 instrument for identification and this may explain why *Ralstonia picketii* was identified from the water specimen as opposed to *Ralstonia mannitolilytica* from clinical specimens. In this case, the banding pattern on ERIC-PCR was identical of the *R.picketii* cultured from water and the clinical *R.mannitoliliytica*. In addition, the antibiogram of the *R.picketii* was also identical to the *R.mannitoliliytica*. It was for these reasons as well as cost saving reasons that we did not send the *R.picketii* isolated for species level identification (i.e. 16S rRNA sequencing).

Following the above findings, we contacted the hospital management and advised that the entire dialysis system be overhauled. The recommendation included changing all the piping in the system as *Ralstonia* species are known to exhibit biofilm formation in plastic water piping [13]. As a minimum, the reverse osmosis pump required urgent repair. It was further advised that the UV lights be checked frequently so as to confirm that they are working at the optimal intensity. It was noted upon enquiry that maintenance of the dialysis water system was lacking with inadequate service records and service contracts. We advised that these should be re-instituted as a matter of urgency.

The hospital management responded positively by repairing the reverse osmosis pump. They also contracted a private company to sterilize the dialysis water system immediately. Another company was contracted to test dialysis water every 3–6 months as per the company policy. These measures proved to be effective as we did not detect any further cases of *Ralstonia mannitolilytica* bacteraemia at the Steve Biko Hospital after this intervention.

*Ralstonia* outbreaks usually persist for prolonged periods [1]. The most likely reason for this is that the source of the outbreak is usually hypothesized and not pinpointed in many studies [1]. Daxboeck et al. (2005) reported isolation of *R. mannitolilytica* in 30 patients attending 15 different wards between February 2002 and March 2004 and in their study, the source of the outbreak was never identified [9]. One of the strengths of this study was the fact that we detected the source of the outbreak and were therefore able to control the outbreak in a matter of 6–8 weeks.

A limitation of this study is that a better method for discriminatory power (such as whole genome sequencing) to determine clonality was not used amongst our isolates. Furthermore we did not determine the antimicrobial resistance genes in the *Ralstonia mannitolilytica* isolates in this study.

## Conclusion

This study highlights the importance of a multidisciplinary team within the hospital to control an outbreak of this nature. The microbiology laboratory team, the treating clinicians, infection control nurses, infectious disease specialists and hospital management play a crucial role in maintaining the safety of patients under the care of their institute. Routine maintenance of the water system is crucial in the prevention of such outbreaks in the future.

## Data Availability

The datasets used and/or analysed during the current study are available from the corresponding author on reasonable request.

## Abbreviations

AST: Antimicrobial susceptibility testing
CLSI: Clinical and Laboratory Standards Institute
DNA: Deoxyribonucleic acid
ERIC-PCR: Enterobacterial Repetitive Intergenic Consensus Polymerase Chain Reaction
PCR: Polymerase chain reaction
rRNA: Ribosomal ribonucleic acid
UV: Ultraviolet
